# Lichen-like anchoring of MoSe_2_ on functionalized multiwalled carbon nanotubes: an efficient electrode for asymmetric supercapacitors†

**DOI:** 10.1039/d0ra06952c

**Published:** 2020-11-04

**Authors:** Swapnil S. Karade, Ajaysing S. Nimbalkar, Jeong-Hyun Eum, Hansung Kim

**Affiliations:** Electrochemical Energy Laboratory, Department of Chemical and Biomolecular Engineering, Yonsei University 50 Yonsei-ro, Seodaemun-gu Seoul-03722 Republic of Korea elchem@yonsei.ac.kr; Korea Research Institute of Chemical Technology Yusong-gu Republic of Korea; University of Science and Technology Daejeon Republic of Korea

## Abstract

In the present study, we have developed a composite electrode of MSNT using a simple and scalable two-step scheme to synthesize a composite electrode material comprising MoSe_2_/multiwalled carbon nanotubes (MoSe_2_/MWCNTs) for supercapacitor applications. First, a MWCNT thin film was deposited on a stainless steel substrate by using a “dip and dry” coating technique. Subsequently, MoSe_2_ was deposited onto the MWCNT thin film using the successive ionic layer adsorption and reaction method. The lichen-like growth of MoSe_2_ on the MWCNT network provided dual charge storage and an effective ion transfer path. The composite electrode of MSNT has been studied systematically with different electrolytes and concentrations of electrolyte. As a result, the MoSe_2_/MWCNT (MSNT) electrode exhibited excellent electrochemical properties such as a specific capacity of 192 mA h g^−1^ and a capacitance retention of 88% after 2000 cycles in 1 M LiCl electrolyte. The results demonstrated the huge potential of the MSNT composite electrode for practical application in supercapacitors. The aqueous symmetric cell fabricated using the MSNT composite as both the anode and cathode showed an energy density of 17.9 W h kg^−1^. Additionally, the energy density improved by designing an asymmetric device of MSNT//MnO_2_ and notably, it reveals two-fold improvement in the energy density compared to a symmetric MSNT cell. The MSNT//MnO_2_-based asymmetric cell exhibited a maximum specific capacitance of 112 F g^−1^ with a high energy density of 35.6 W h kg^−1^.

## Abstract 2

Simple and scalable chemical synthesis approach to develop a MoSe_2_/MWCNTs composite thin film electrode for a highly efficient asymmetric supercapacitor cell.

## Graphical abstract

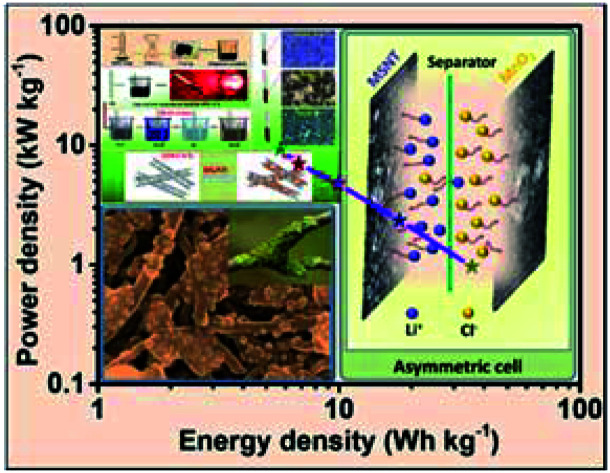

## 1. Introduction

Carbon-based materials such as carbon nanotubes (CNTs), graphene, reduced graphene oxide (rGO), and activated carbon (AC) demonstrate electrochemical double-layer capacitive behavior. These materials exhibit large surface areas, less degradation, high stability, and good electrical conductivity. However, carbon-based materials suffer from low capacitance.^1,2^ Nanostructured inorganic and organic materials such as metal oxides/hydroxides, metal chalcogenides, and conducting polymers show pseudocapacitive charge storage behavior.^3–6^ Recently, metal phosphides are coming into the picture of supercapacitive materials with a superior electrochemical performance.^7–9^ Zhou *et al.*^7^ developed a pseudocapacitive Ni_2_P nanosheet directly grown on Ni foam *via* phosphorization. The Ni_2_P electrode shows remarkable specific capacitance of 2141 F g^−1^ with a good retention of 1109 F g^−1^ even at a high current density of 83.3 A g^−1^. Further, an asymmetric device of Ni2P as a positive electrode and activated carbon (AC) as a negative electrode shows significant results with a specific capacitance of 96 F g^−1^ and energy density of 26 W h kg^−1^. Zhang *et al.*^8^ fabricate a high-performance asymmetric supercapacitor using bimetallic phosphide of Co_*x*_Ni_1−*x*_P on carbon nanofibers (CNF) as a positive electrode and AC as negative electrode. It reveals significant electrochemical properties with a potential window of 1.4 V, specific capacitance of 85 F g^−1^ and energy density of 32.2 W h kg^−1^. Also, Zhang *et al.*^9^ reported CoP/CNF prepared by using chemical reduction and used as a negative electrode to made a high-performance asymmetric supercapacitor with a NiP/CNF as a positive electrode. Notably, the asymmetric device delivers maximum specific capacitance of 164 F g^−1^ with a high energy density of 56 W h kg^−1^ and long-term stability up to 5000 cycles. As compared to carbon-based materials, these materials show high capacitance; however, they degrade during the charge–discharge process, and hence show poor cycling stability.^3–6,10,11^ Therefore, various nanocomposites have been prepared in order to overcome the limitations and improve the electrochemical performance of single materials.

In the last decade, metal dichalcogenides have gained significant attention in the field of energy storage and conversion owing to their chemical, physical, optical, electrical, and mechanical properties.^12–16^ In addition, their layered graphene-like structure, higher electrical conductivity than that of metal oxides, multivalent oxidation states, earth abundance, and nontoxicity make them promising candidates for commercial applications.^5,17,18^ Molybdenum- and tungsten-based chalcogenides are two-dimensional (2D) materials with nanosheets and flower-like nanostructures.^19,20^ Soon and Loh *et al.* demonstrated the use of transition metal chalcogenide-based materials for supercapacitor applications. In 2007, MoS_2_ nanowall films with an electrochemical double-layer capacitance were reported.^21^ Since then, various developments have been made in the preparation of molybdenum- and tungsten-based chalcogenides for supercapacitor applications.^22–30^

At present, numerous reports are available on the use of MoS_2_, MoSe_2_, and MoTe_2_ as supercapacitor electrodes. Balasingam *et al.* synthesized few-layered MoSe_2_ nanosheets for supercapacitor applications by using a hydrothermal method. When used as an electrode, these nanosheets exhibited a significant specific capacitance of 199 F g^−1^ at a scan rate of 2 mV s^−1^ in 0.5 M H_2_SO_4_ as the electrolyte.^31^ The authors extended their work by developing complex MoSe_2_/rGO nanosheets and obtained a higher specific capacitance of 211 F g^−1^ at the scan rate of 2 mV s^−1^.^32^ In addition, Huang *et al.* reported a simple hydrothermal strategy for the synthesis of MoSe_2_ and its direct deposition on Ni foam. The resulting MoSe_2_/Ni foam electrode showed a high capacitance of 744 F g^−1^ and the faradaic charge storage behavior in 6 M KOH as the electrolyte.^33^ A binder-free approach based on the highly efficient electrodeposition method was successfully utilized for the deposition of 2D MoSe_2_ on a Ni foam.^34^ The resulting electrode showed a battery-type charge storage behavior with a capacity of 548 mA h g^−1^. Furthermore, a highly porous net-like acetylene black-supported MoSe_2_ composite electrode was synthesized by using the hydrothermal method. The MoSe_2_-acetylene black composite electrode showed an outstanding specific capacitance of 2020 F g^−1^ and an excellent cyclic retention of 107% after 1500 cycles.^35^ In addition to bare MoSe_2_ and carbon–MoSe_2_ composites, high-performance heterostructural composites such as NiSe@MoSe_2_ and CoNi_2_S_4_–graphene–MoSe_2_ have also been reported.^36,37^

Developing a hybrid nanostructure with a carbon matrix is an efficient approach to improve the performance of MoSe_2_ electrodes. Owing to their one-dimensional (1D) structure, large surface area, good chemical stability, and mechanical flexibility, multiwalled carbon nanotubes (MWCNTs) are considered as the most promising matrix materials for supporting the host materials.^38–40^ Furthermore, the 1D structure of MWCNTs provides good electrical conductivity and efficient electron transfer pathways. In addition, the MWCNT surface reduces the aggregation of the host material. Therefore, in this study, we developed a simple, scalable, and binder-free two-step chemical process. A MWCNT thin film was fabricated using the “dip and dry” coating method followed by the deposition of MoSe_2_ using the successive ionic layer adsorption and reaction (SILAR) method. The structure and morphology of the MoSe_2_/MWCNT (MSNT) composite thin film were analyzed. The charge storage behavior of the composite thin film in various electrolytes (with different concentrations) was investigated. The MSNT composite electrode showed enhanced electrochemical properties compared to the pristine MoSe_2_ electrode, suggesting that the MSNT composite thin film possesses great potential for application as an electrode material for supercapacitor devices. The symmetric and asymmetric cells demonstrated good electrochemical performance.

## 2. Experimental

### 2.1. Materials

Stainless steel (SS) strips (grade 306) were used as the substrate. All the commercially available analytical reagents, *i.e.*, ammonium heptamolybdate [(NH_4_)_6_Mo_7_O_24_], citric acid (C_6_H_8_O_7_), sodium borohydride (NaBH_4_), Na_2_SO_3_ (sodium sulfite), and selenium (Se) metal powder were purchased from Sigma-Aldrich and used without further treatment. Chemical vapor-deposited MWCNTs were purchased from Nano Amor (Houston, USA). To prepare all the solutions, double-distilled water (DDW) was used as the solvent in all the experiments. The sodium selenosulfite (Na_2_SeSO_3_) precursor was prepared using a previously optimized procedure.^41^ A bath containing 5 g of Se mixed with 15 g of Na_2_SO_3_ in 200 mL of DDW was refluxed at 90 °C for 9 h to obtain a clear solution of Na_2_SeSO_3_. The prepared Na_2_SeSO_3_ solution was then stored in an airtight container and used as the Se source.

### 2.2. Synthesis of the MoSe_2_/MWCNTs composite thin film

First, functionalized MWCNTs were deposited on the SS substrate.^6^ In brief, the commercially available MWCNTs were purchased from Nano Amor (Houston, USA) and were then refluxed with H_2_O_2_ at 90 °C for 48 h to remove amorphous carbon derivatives and generate oxygenated functional groups. The obtained filtrate was repeatedly rinsed with DDW and dried overnight in an oven at 60 °C. The functionalized MWCNTs (0.250 g) were mixed in a 50 mL solution of DDW and the Triton X-100 surfactant (Tritonx, 100 : DDW = 1 : 100). The obtained solution was sonicated for 1 h to obtain a stable dispersion. A mirror-polished SS substrate was vertically immersed in the solution for 10 s for the adsorption of the MWCNTs onto the SS substrate; the SS was then dried under an infrared (IR) lamp to evaporate the solvent. This process was repeated 12 times to obtain a terminal thickness of MWCNTs on the SS and to form adequate nucleation sites with a web-like porous nanonetwork and large surface area for the deposition of MoSe_2_.

In the second step, the MoSe_2_ thin film was deposited by alternately immersing the SS and precoated SS/MWCMT substrates in separate cationic and anionic precursors at ambient temperature. The first beaker consisted of 20 mL of 25 mM ammonium heptamolybdate as the source of Mo ions with 5 mL of 1 M citric acid and 1 mL of 1% sodium borohydride as reducing agents. The second beaker was filled with DDW to remove the loosely bound complexed cation species. The third beaker contained 25 mL of a 0.25 N Na_2_SeSO_3_ solution with 1 mL of a 1% sodium borohydride solution as the reducing agent. The fourth beaker was filled with DDW for the second rinsing to remove the loosely bound molecules and unreacted ion species. The SS and precoated MWCNT substrates were sequentially immersed in the four beakers for 20, 10, 20, and 10 s, representing one complete SILAR cycle. Twenty consecutive SILAR cycles were sufficient to obtain a MoSe_2_ thin film with a desired thickness. The dipping area 3 cm × 3 cm of the stainless-steel sample was coated by MoSe_2_ and MSNT thin films. The adhesion of MWCNT thin film is quite good enough to stable during SILAR process. Fig. S1† shows deposition of MSNT composite thin film which clearly shows MWCNT has good adhesion on SS substrate which does not lose a physical contact during SILAR process (ESI S1†). Basically, weight difference method was used to measure the loading of MoSe_2_ and MSNT thin films.

1 *m* = *m*_2_ − *m*_1_

**Fig. 1:**
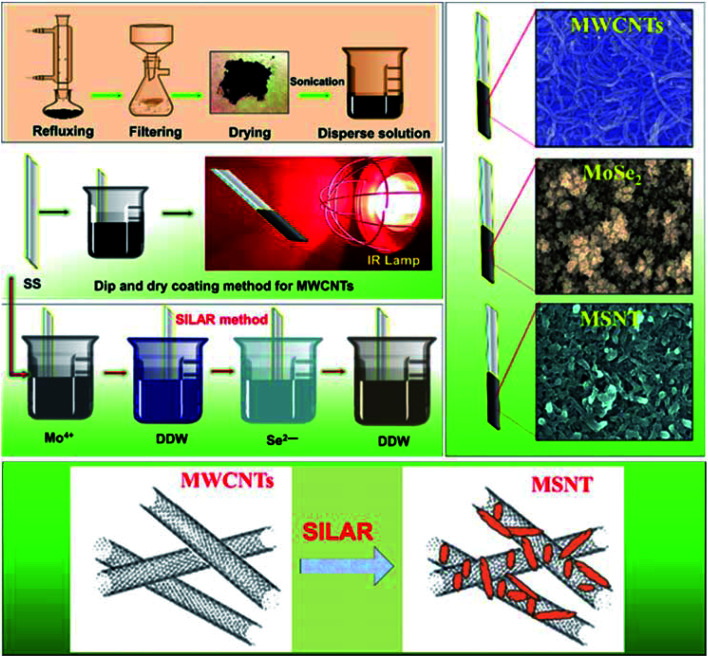
Schematic of the simple and scalable synthesis of the MWCNT, MoSe_2_, and MSNT thin films.

In which, ‘*m*’ is loading of active materials, ‘*m*_1_’ is weight of substrate before deposition and ‘*m*_2_’ is weight of substrate after deposition of thin films. Fig. 1 shows the schematic of the preparation of the MWCNTs, MoSe_2_, and MSNT thin films. The samples were characterized using various analytical techniques (ESI S2†).

### 2.3. Electrochemical setup

The prepared MoSe_2_ and MSNT electrodes were used as the working electrodes and a Pt wire and Ag/AgCl as the counter and reference electrodes, respectively. The electrochemical properties of the electrodes were investigated using a potentiostat/galvanostat (PARSTAT 4000, Princeton Applied Research, USA). The mass loading played an important role in the evaluation of the specific electrochemical parameters of the electrodes. The weights of the MoSe_2_ and MSNT electrodes were 0.23 and 0.40 mg cm^−2^, respectively.

## 3. Results and discussion

### 3.1. Reaction kinetics

Lichen-like MoSe_2_ nanoparticles were deposited on the MWCNT surface *via* the electrostatic adsorption of cations and their reaction with anions. Initially, (NH_4_)_6_Mo_7_O_24_ was used as the Mo source, which dissociated in DDW to form heptamolybdate ions.^42^

2 (NH_4_)_6_Mo_7_O_24_ → 6NH_4_^+^ + Mo_7_O_24_^6−^

After the addition of citric acid, these heptamolybdate ions formed a complex with citrate ions [C_3_H_5_O(COO)_3_^3−^], as shown below :^43^

3 Mo_7_O_24_^6−^ + 2C_3_H_5_O(COO)_3_^3−^ → [Mo–2C_3_H_5_O(COO)_3_] + 12O_2_

NaBH_4_ (reducing agent) reduced the Mo^6+^ ions in the precursor to Mo^4+^ according to the following reaction:^39,41,43^

4 [Mo–2C_3_H_5_O(COO)_3_] + 2BH_4_^−^ + 2H_2_O → Mo^4+^ + 2C_6_H_8_O_7_ + 2BH_3_ + O_2_↑

The bare SS and MWCNT-coated SS substrates were immersed in the aforementioned solution for the adsorption of Mo^4+^ ions.

The dissociation of Na_2_SeSO_3_ produced Na^+^and SeSO_3_^2−^, which were further reduced in the presence of NaBH_4_ (reducing agent) to form Se^2−^ ions.

5 SeSO_3_^2−^ + 2BH_4_^−^ → Se^2−^ + 2BH_3_ + H_2_↑ + SO_3_^2−^

Finally, the Mo^4+^ ion-adsorbed bare SS and MWCNT-coated SS substrates were immersed in the aforementioned solution containing Se^2−^ ions to form MoSe_2_.

6 Mo^4+^ + 2Se^2−^ → MoSe_2_

The aforementioned process was repeated several times by immersing the bare SS and MWCNT-coated SS substrates in the respective solutions (Mo^4+^ and Se^2−^) to deposit a uniform and well-adhering thin layer of MoSe_2_ on the substrates.

### 3.2. Structural analysis

**Fig. 2:**
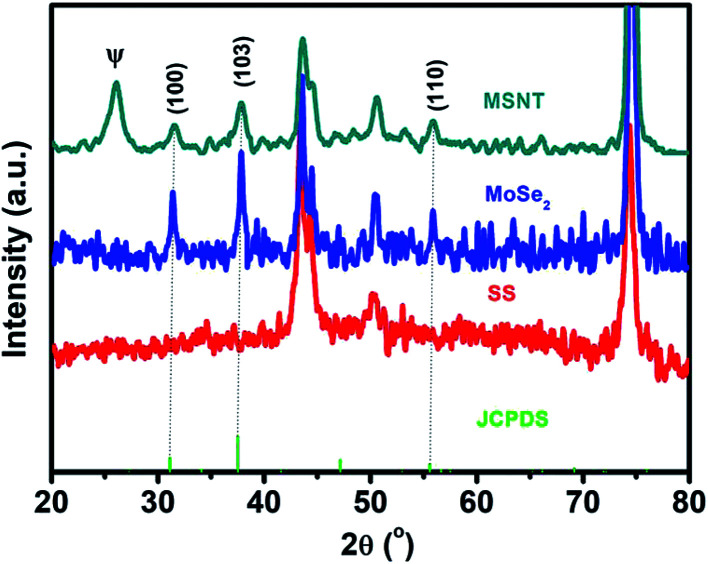
XRD patterns of the MoSe_2_ and MSNT thin films.

**Fig. 3:**
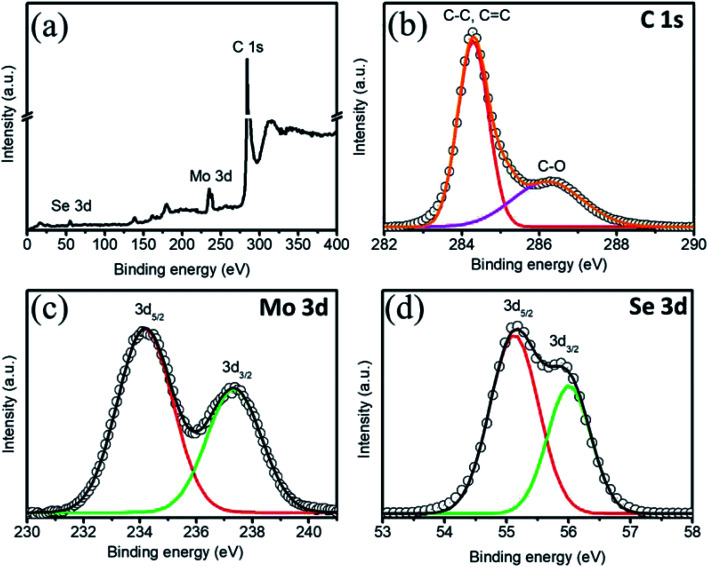
(a) XPS profile of the MSNT composite. Core-level (b) C 1s, (c) Mo 3d, and (d) Se 3d XPS profiles.

The structures of the MSNT composite and MoSe_2_ thin films were analyzed using wide-angle X-ray diffraction (XRD) (Fig. 2). The films showed major diffraction peaks at 2*θ* = 31.4°, 37.8°, and 55.8° corresponding to the (100), (103), and (110) planes of hexagonal MoSe_2_ (JCPDS No. 87-2419), respectively, while the other peaks listed in the JCPDS card could not be observed because of their low intensity and the relatively high-intensity peaks of the SS substrate. Furthermore, the broad peak observed at 2*θ* = 26° in the XRD pattern of the MSNT thin film denoted by *Ψ* was attributable to the characteristic peak of the graphitic carbon of the MWCNTs. The surface elemental compositions of the thin films were analyzed using X-ray photoelectron spectroscopy (XPS). Fig. 3a shows the XPS survey profile of the MSNT composite film. As can be observed from the figure, the film showed peaks corresponding to the Se 3d, Mo 3d, and C 1s orbitals. This further confirmed the formation of the MSNT composite thin film. Fig. 3b shows the core C 1s profile of the film. Two prominent peaks were observed at the binding energies of 284.1 and 286.3 eV corresponding to the graphitic carbon of the MWCNTs and the carbon–oxygen bonds, respectively. The narrow-scan Mo 3d XPS profile of the film (Fig. 3c) showed two distinct peaks at 234.2 and 237.3 eV, indicating that the oxidation state of Mo was 4+.^32,39,44–46^ In addition, Fig. 3d shows the Se 3d XPS profile of the film. The Se 3d peak of the composite film could be deconvoluted into two peaks at 55.2 and 56 eV, indicating that the oxidation state of Se was 2−.^32,36^

### 3.3. Surface morphology

**Fig. 4:**
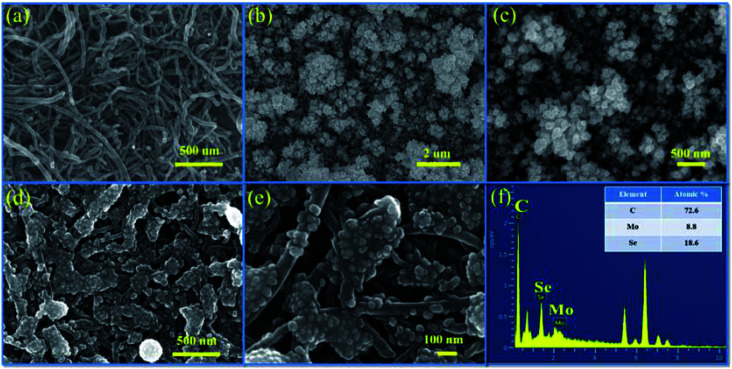
FESEM images of (a) MWCNTs, (b and c) MoSe_2_, and (d and e) the MSNT composite thin film at different magnifications; and (f) EDS mapping of the MSNT composite thin film.

The surface microstructure of a material significantly affects its charge storage capacity. Fig. 4a shows the microscopic image of the surface of the MWCNT thin film deposited on the SS substrate. Owing to its uniform network-like structure, the MWCNT thin film showed a large surface area and 1D conduction path for electron transfer. The successive cation adsorption and anion reaction resulted in the colonial growth of MoSe_2_ nanoparticles on the MWCNT thin film, as shown in Fig. 4b and c. Electrodes with a porous structure show large surface area and numerous active sites for interaction with electrolyte ions, and hence offer significant storage ability.^47^ In the MSNT composite thin film, the lichen-like growth of MoSe_2_ on the MWCNTs (Fig. 4d and e) reduced the ion diffusion path length and exhibited strong interfacial conjugation. In addition, the porous structure of the MSNT composite provided large effective surface area for the interaction of the electrolyte ions during the electrochemical process, which in turn improved the electrochemical performance of the composite. Fig. 3f shows the energy-dispersive spectroscopy (EDS) mapping of the MSNT composite thin film, which revealed that the film consisted of 72.6% C, 8.8% Mo, and 18.6% Se, confirmed the formation of the MoSe_2_/MWCNT composite structure.

**Fig. 5:**
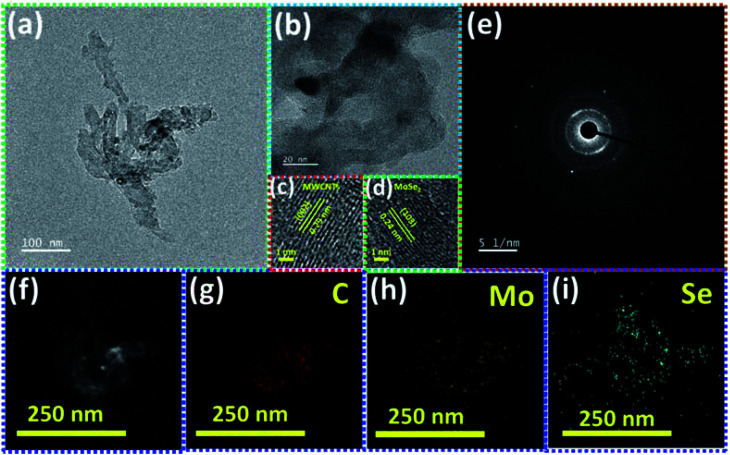
(a) TEM and (b–d) high-resolution TEM images of the MSNT film with the corresponding enlarged view of the lattice fringes, (e) SAED pattern and (f–i) EFTEM mapping of the MSNT film.

The transmission electron microscopy (TEM) image of the MSNT composite is shown in Fig. 5a. It clearly reveals the lichen-like growth of MoSe_2_ nanoparticles on the MWCNTs. The oxygenated functional groups generated during the reflux of the MWCNTs with H_2_O_2_ acted as active sites for the growth of MoSe_2_ on the MWCNTs. Fig. 5b shows the high-magnification TEM image of the MSNT composite film. The lattice fringes with the interplanar spacings of 0.29 nm and 0.24 nm correspond to the (002) plane of graphitic carbon in the MWCNTs and the (103) plane of the hexagonal MoSe_2_ crystal, respectively (Fig. 5c and d). The selected area electron diffraction (SAED) pattern of the composite film showed concentric rings, indicating its polycrystalline nature (Fig. 5e). The elemental distribution of the MSNT composite sample was analyzed by obtaining its energy-filtered TEM (EFTEM) images (Fig. 5f–i), which confirmed the formation of the MSNT composite.

### 3.4. Electrochemical analysis

**Fig. 6:**
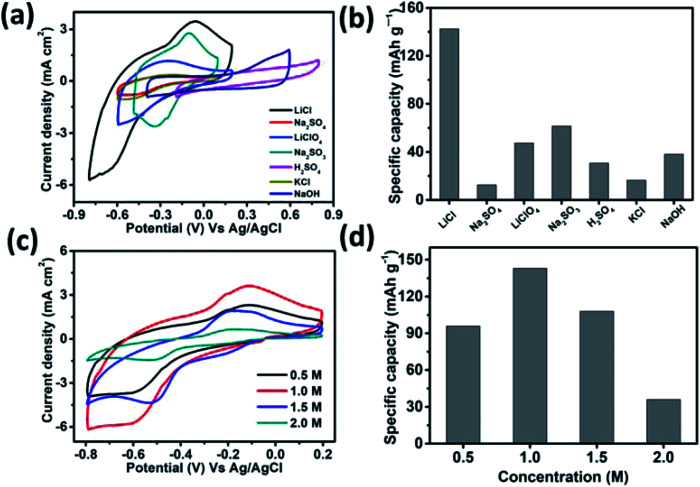
(a) CV curves of the MSNT composite electrode in various electrolytes at a scan rate of 20 mV s^−1^, (b) specific capacity values for various electrolytes, (c) effect of the LiCl electrolyte concentration on the CV curves of the MSNT composite electrode at a scan rate of 20 mV s^−1^, and (d) the effect of the LiCl concentration on the specific capacity of the MSNT electrode.

The performance of the MSNT electrode was analyzed using a conventional three-electrode system in a suitable electrolyte with an optimum concentration. Aqueous LiCl, Na_2_SO_4_, LiClO_4_, Na_2_SO_3_, H_2_SO_4_, KCl, and NaOH (1 M) were used as the electrolytes at a constant scan rate of 20 mV s^−1^ (Fig. 6a) under Ag/AgCl as reference electrode. The low thermodynamic stability of water is a major disadvantage for aqueous electrolytes.^48^ The thermodynamic stability of water under the potential limit varies as a function of the pH of the electrolyte solution. Therefore, the cyclic voltammetry (CV) curves of the MSNT electrode showed different potential windows for different electrolytes (Fig. 6a). Fig. 6b shows the specific capacitance values of the electrodes in the different electrolytes used. The aqueous 1 M LiCl electrolyte exhibited the highest specific capacity of 142 mA h g^−1^ at the scan rate of 20 mV s^−1^. It is well-known that the specific capacity of an electrode is a charge-dependent quantity, and the electrolyte concentration determines the quantity of charges. Hence, the effect of the LiCl concentration (0.5–2 M) on the CV performance of the MSNT composite electrode at a fixed scan rate of 20 mV s^−1^ was investigated (Fig. 6c). Fig. 6d shows the effect of the LiCl concentration on the specific capacity of the MSNT composite electrode. As can be observed from the figure, the specific capacity of the electrode increased with an increase in the LiCl concentration from 0.5 to 1 M. With a further increase in the LiCl concentration, the specific capacity of the electrode decreased. This is because at higher LiCl concentrations, the channels within the electrode layer were effectively consumed by the electrolyte ions. The ion activity decreased with an increase in the LiCl concentration because of the deceleration of the water hydration reaction, which reduced the mobility of the electrolyte ions.^6,46^ The optimum LiCl concentration was found to be 1 M. This concentration was used for the electrochemical analyses of the MoSe_2_ and MSNT thin film electrodes.

**Fig. 7:**
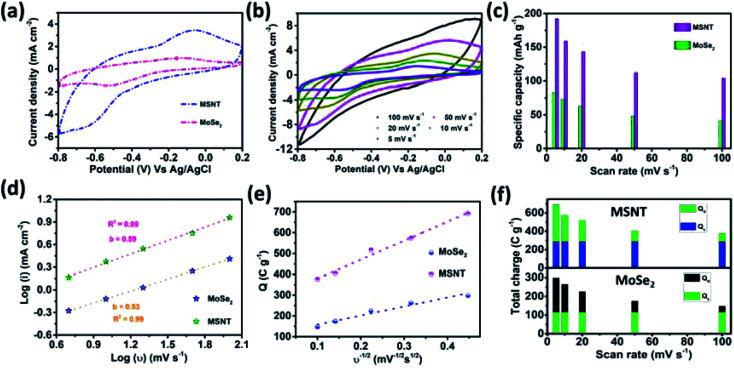
(a) CV curves for the MoSe_2_ and MSNT electrodes obtained at a constant scan rate of 20 mV s^−1^, (b) CV curves for the MSNT electrode at the scan rates ranging from 5 to 100 mV s^−1^, and (c) specific capacity values of the MoSe_2_ and MSNT electrodes at different scan rates, (d) determination of the *b*-value from the log(*i*) *vs.* log(*ν*) plots of the electrodes, (e) total charge stored *vs.* reciprocal square root of scan rate (*Q vs. ν*^−1/2^) plots of the electrodes, and (f) contribution of the surface capacitive (*Q*_s_) and diffusion-controlled (*Q*_d_) charges to the total charge (*Q*_t_).

The CV curves of the MoSe_2_ and MSNT electrodes at the fixed scan rate of 20 mV s^−1^ and over the potential range of −0.8–0.2 V *vs.* Ag/AgCl are shown in Fig. 7a. The MSNT electrode showed higher current distribution than the MoSe_2_ electrode because of the synergistic effect and good interfacial conjugation between MoSe_2_ and the MWCNTs. In the 1 M aqueous LiCl electrolyte, the MoSe_2_ and MSNT electrodes showed a faradaic charge storage behavior characterized by two distinguishable oxidation and reduction peaks. Fig. 7b shows the CV curves of the MSNT composite electrode at different scan rates ranging from 5 to 100 mV s^−1^. The CV curves of the bare MoSe_2_ electrode is shown in the ESI S3 (Fig. S2†). The current distribution under the CV curves of both the electrodes increased with an increase in the scan rate, indicating the capacitive nature of the electrodes. In both the electrodes, two charge storage processes occurred during the CV cycling, as indicated by the shape of the CV curves of the electrodes.^49^ One of these processes could be attributed to the surface adsorption at the interface between the electrode and the electrolyte. A double layer was formed at the electrode/electrolyte interface.

7 (MoSe_2_)_surface_ + *x*Li^+^ + *x*e^−^ ⇌ (Li_*x*_–MoSe_2_)_surface_

The second process corresponded to the reversible faradaic reaction based on the intercalation/deintercalation of Li-ion species.

8 MoSe_2_ + *x*Li^+^ + *x*e^−^ ⇌ Li_*x*_MoSe_2_

Thus, the electrochemical properties of these electrodes were achieved by utilizing the overall mass of the electrode material through the surface adsorption and intercalation processes. Fig. 7c shows the correlation between the gravimetric capacity and scan rate for both the electrodes. The MSNT composite electrode showed the maximum capacitance, which was more than two times higher than that of the bare MoSe_2_ electrode. The capacitance decreased with an increase in the scan rate. At higher scan rates, the ion concentration on the surface of the electrode decreased with an increase in the current, and the ions diffused slowly during the charge–discharge process. In addition, a decrease in the capacitance was observed with an increase in the scan rate because the inner active sites of the electrode could not be sufficiently utilized and could not sustain the complete intercalation/deintercalation process at high scan rates because of time constraints.^50^ The unique nanostructure with lichen-like MoSe_2_ on the 1D porous MWCNT network facilitated rapid ion/electron transfer for the intercalation/deintercalation of ions and induced a strong synergistic effect, thus improving the electrochemical properties of the MSNT composite electrode.^51^ Thus, a very high capacitance of 690 F g^−1^ was obtained at a low scan rate of 5 mV s^−1^ because of the effective utilization of all the active sites. The charge storage mechanisms of the electrodes were examined by obtaining their CV curves. The relationship between the current and scan rate can be expressed as follows.^52^

9 *I* = *aν*^*b*^

where, ‘*a*’ and ‘*b*’ are the adjustable parameters calculated from the log(*I*) *vs.* log(scan rate) plot shown in Fig. 7d. The *b*-value is important to investigate the charge storage kinetics of an electrode. A *b*-value of 1 denotes a capacitive current, while *b* = 0.5 implies that the current generated during charge storage is diffusion-controlled. As can be observed from Fig. 7d, the *b*-values for the anodic peak currents of the MoSe_2_ and MWCNTs electrodes were 0.53 and 0.59, respectively. This indicates that both the electrodes showed diffusion-controlled charge storage kinetics. The total charge (*Q*_t_) of the electrodes was contributed by the surface capacitive (*Q*_s_) and diffusion-controlled (*Q*_d_) kinetics occurring at the electrode/electrolyte interface.

10 *Q*_t_ = *Q*_s_ + *Q*_d_

Considering the semi-infinite linear diffusion, the *Q*_s_ of the electrodes could be determined by plotting the graph of *Q*_t_ as a function of the reciprocal square root of the scan rate, as shown in Fig. 7e.

11 *Q*_t_ = *Q*_s_ + *cv*^−1/2^

The surface capacitive and diffusion-controlled charges contributed to the total charge stored by the MoSe_2_ and MSNT electrodes (Fig. 7f), indicating that the charge storage mechanisms of the electrodes were mainly governed by their redox reactions.

**Fig. 8:**
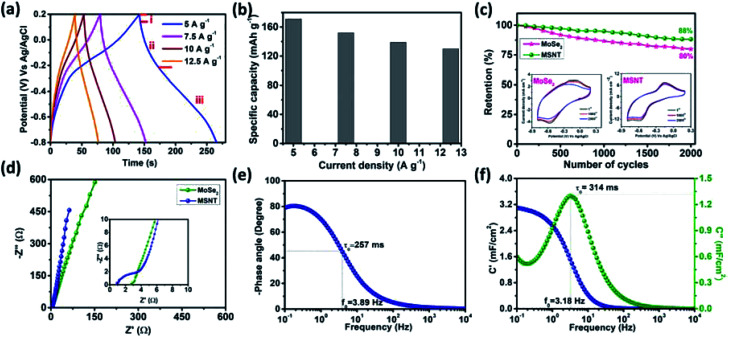
(a) Charge–discharge profiles of the MSNT electrode at current densities ranging from 2 to 5 mA cm^−2^, (b) specific capacity of the MSNT electrode as a function of the current density (from 5–12.5 A g^−1^), (c) cyclic retention of the MoSe_2_ and MSNT electrodes over 2000 cycles at a fixed scan rate of 100 mV s^−1^; the inset shows the change in the CV curves after cycling, (d) Nyquist plot for the MoSe_2_ and MSNT electrodes, (e) Bode plot of the MSNT electrode, and (f) real and imaginary parts of the capacitances (*C*′ and *C*′′) *vs.* frequency plot for the MSNT electrode.

The correlation between the potential of the electrodes and time at the constant current densities of 2–5 mA cm^−2^ was investigated by obtaining their galvanostatic charge–discharge (GCD) curves. Fig. 8a shows the GCD curves of the MSNT composite electrode. The deviated triangular shape of the curves was due to the faradaic charge storage behavior of the MSNT electrode. In addition, the discharge curve of the electrode could be divided into three parts (Fig. 8a)—(I) an initial potential drop due to the internal resistance of the electrode, (II) a linear deviation because of the charge separation due to the non-faradaic reaction at the interface between the electrode and the electrolyte, and (III) an inclined aberration indicating the faradaic charge storage caused by the reversible intercalation/deintercalation of Li^+^ ions.^53–55^Fig. 8b shows the specific capacities of the MSNT electrode at various current densities, as calculated from its GCD curves. The electrode showed a maximum specific capacity of 171 mA h g^−1^ at 5 A g^−1^ and could retain a specific capacity of 130 mA h g^−1^ at a high current density of 12.5 mA h g^−1^.

Long-term electrochemical stability is a prerequisite for realizing the practical applications of an electrode. The stability of the MSNT electrode was investigated over 2000 CV cycles at a fixed scan rate of 100 mV s^−1^ in a 1 M aqueous LiCl electrolyte. Fig. 8c shows the capacity retention of the electrodes over 2000 CV cycles. The MSNT composite electrode showed a capacity retention of 88%, which was higher than that of the bare MoSe_2_ electrode (80%). The improved stability of the MSNT electrode compared to that of the bare MoSe_2_ electrode can be attributed to the strong synergistic effect and interfacial conjugation between MoSe_2_ and the MWCNTs.

Electrochemical impedance spectroscopy (EIS) was used to analyze the capacitive and resistance parameters of the electrodes over the frequency range of 0.1 mHz to 10 kHz at the bias potentials of 0 and 10 mV. Fig. 8d shows the imaginary (*Z*′′) and real (*Z*′) parts of the impedance of the MoSe_2_, and MSNT electrodes. The Nyquist plots of the electrodes showed an intercept with a semicircular arc in the high-frequency region and a straight line in the low-frequency region. The non-zero intercept on the real-axis indicates the equivalent series resistance (*R*_s_), which is the combination of the solution resistance, internal resistance of the active electrode, and the resistance between the active electrode and the current collector. The semicircle corresponds to the faradaic reversible reaction that occurs during the charge storage and the diameter of the semicircle indicates the charge transfer resistance (*R*_ct_) associated with the electrode parallel to the double-layer capacitor (*C*_dl_). The *R*_s_ and *R*_ct_ values of the MoSe_2_, and MSNT electrodes were 0.95 and 0.41; and 2.71 and 2.01 Ω respectively. These considerably small *R*_s_ and *R*_ct_ values indicate an intimate interfacial contact between the electrode and the Li^+^ ions in the electrolyte.^55,56^

The frequency-dependent phase angles of the electrodes were determined by obtaining their Bode plots (Fig. 8e). The phase angle of the MSNT electrode increased with a decrease in the frequency and became approximately −90° as the frequency approached the minimum value. This indicates that the MSNT electrode showed a capacitive behavior of the MSNT electrode.^57–59^ Additionally, the steeply oblique line at low frequency region suggests the MSNT electrode reveals rapid response of electrolyte ion diffusion during charge–discharge.^59^ Furthermore, the ideal capacitive behavior of the electrode could be assessed by determining the frequency at which the phase angle crossed 45°.^58^ The MSNT electrode exhibited a frequency of 3.89 Hz when the phase angle crossed 45°, indicating a capacitive behavior with rapid frequency response. Furthermore, the capacitive performance of the electrode could be evaluated by considering the real and imaginary parts of its capacitance with respect to the frequency:

12 *C*(*ω*) = *C*′(*ω*) − *jC*′′(*ω*)

13
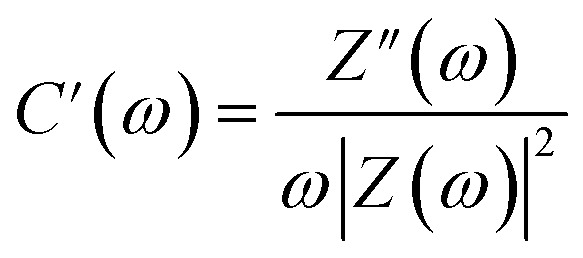
14
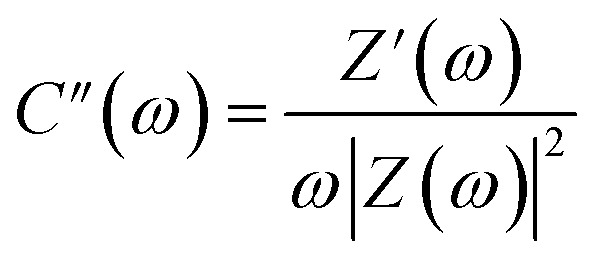
where |*Z*(*ω*)| = *Z*′(*ω*) + *Z*′′(*ω*) is the complex impedance; *ω* = 2π*f*, where *f* is the frequency; *Z*′and *Z*′′are the real and imaginary parts of the impedance in the Nyquist plot, respectively; *C*′(*ω*) is the real accessible capacitance of the electrode; and *C*′′(*ω*) is the imaginary part of the capacitance corresponding to the energy loss due to the irreversible process of the electrode. The frequency-dependence of the real and imaginary parts of the capacitance (*C*′ and *C*′′) of the MSNT electrode is shown in Fig. 8f. The plot showed relaxation-type dispersions, where the real part of the capacitance reached a maximum value at lower frequencies and decreased with an increase in the frequency. This indicates that the electrode acted as an open circuit in the low-frequency region when the utilization of the electrode material was the maximum. When the utilization of the electrode material was the least, the electrode acted as a short circuit in the high-frequency region.^60,61^ The imaginary part of the capacitance showed a maximum value at a frequency of 3.18 Hz, which is comparable to that obtained in the Bode plot at the phase angle of 45°. The parameter *f*_0_ is the characteristic frequency at which the time required to lose all the energy of the electrode is minimal. The term is known as the relaxation time constant and can be calculated using the following equation: *τ*_0_ = 1/*f*_0_. The *τ*_0_ value of the hybrid MSNT electrode was 314 ms, which was much lower than that of carbon electrodes such as rGO (430 ms),^62^ hybrid graphene (392 ms),^63^ and AC (700 ms).^64^ This indicates that the hybrid MSNT electrode showed excellent charge–discharge rate.

### 3.5. Symmetric and asymmetric supercapacitor cell

**Fig. 9:**
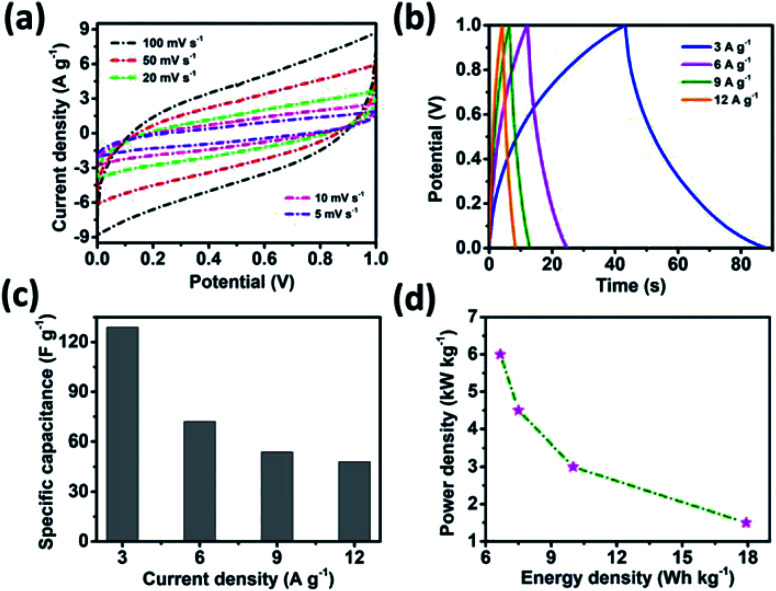
Electrochemical characterizations of the MSNT//MSNT symmetric cell. (a) CV curves at different scan rates ranging from 5 to 100 mV s^−1^, (b) GCD curves at various current densities (3–12 A g^−1^), (c) specific capacitance values from the GCD plot, and (d) Ragone plot of the MSNT//MSNT symmetric cell.

The potential of the MSNT composite electrode for supercapacitor applications was evaluated by fabricating a symmetric supercapacitor cell. The symmetric cell was fabricated using the MSNT composite as both the cathode and anode separated by a Whatman filter paper soaked with 1 M aqueous LiCl as the electrolyte. Fig. 9a shows the CV curves of the cell at scan rates in the range of 5–100 mV s^−1^ over the potential window of 0–1 V. These curves indicated that the cell showed a pure capacitive behavior. The shape of the CV curves remained almost constant at different scan rates, indicating that the cell showed significant reversibility. Furthermore, the GCD plots of the MSNT symmetric cell at different current densities (3–12 A g^−1^) are shown in Fig. 9b. The MSNT symmetric cell delivered a maximum specific capacitance of 129 F g^−1^ at 3 A g^−1^ (Fig. 9c). The Ragone plots of the cell showing the correlation between its specific energy and specific power are shown in Fig. 9d. The MSNT symmetric cell delivered a significant specific energy of 17.9 W h kg^−1^ at a specific power of 1.5 kW kg^−1^. Table 1 lists the specific capacitance, specific energy, and specific power of the MSNT symmetric cell.

**Table 1:** Electrochemical parameters of the MSNT//MSNT symmetric cell

Current density (A g^−1^)	Specific capacitance (F g^−1^)	Energy density (W h kg^−1^)	Power density (W kg^−1^)
2	129	17.9	1500
6	72	10	3000
9	54	7.5	4500
12	48	6.7	6000

**Fig. 10:**
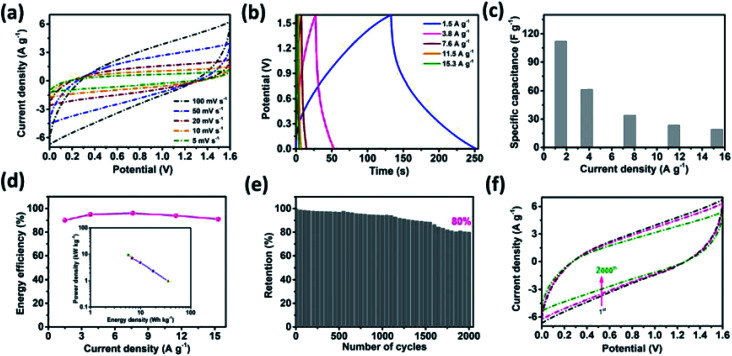
Electrochemical characterizations of the MSNT//MnO_2_ asymmetric cell. (a) CV curves at different scan rates ranging from 5 to 100 mV s^−1^, (b) GCD curves at various current densities (from 1.5 to 15.3 A g^−1^), (c) specific capacitance values from the GCD plot, and (d) energy efficiency *vs.* current density curves; the inset shows the Ragone plot of the cell, (e) capacitance retention performance of the cell over 2000 cycles, and (f) charge of the cell after 2000 cycles at a fixed scan rate of 100 mV s^−1^.

An asymmetric supercapacitor cell was employed to achieve a high energy density over a wide working potential window. A MnO_2_ thin film was used as the positive electrode to fabricate the MSNT//MnO_2_ asymmetric supercapacitor.^65^ The charge contribution of the positive (MnO_2_) and negative (MSNT) electrodes (CV curves) and the potential stability of the asymmetric cell (GCD plots) are shown in Fig. S3 (ESI S4†). The CV curves of the cell at different scan rates (5–100 mV s^−1^) are shown in Fig. 10a. The asymmetric cell showed a wide potential window of 1.6 V with no significant polarization. In addition, the shape of the CV curves remained unchanged at high scan rates, indicating the excellent rate capability of the fabricated asymmetric cell. Fig. 10b shows the GCD plots of the MSNT//MnO_2_ asymmetric cell at different current densities ranging from 1.5 to 15.3 A g^−1^. The specific capacitance of the cell is shown in Fig. 10c. The maximum specific capacitance of 112 F g^−1^ was achieved at the current density of 1.5 A g^−1^. The cell retained a specific capacitance of 19 F g^−1^ at 15.3 A g^−1^. The inset of Fig. 10d shows the Ragone plot (specific energy *vs.* specific power) of the cell. The MSNT//MnO_2_ cell showed a maximum specific energy of 35.6 W h kg^−1^ at the power density of 964 W kg^−1^ and maintained a significant specific energy of 5.7 W h kg^−1^ even at the high power density of 9327 W kg^−1^. The asymmetric cell showed a significantly higher specific energy than the symmetric MSNT supercapacitor cell because of its wide working potential. The MSNT//MnO_2_ asymmetric cell could retain up to 90–96% of its energy efficiency (Fig. 10d). This indicated that the asymmetric cell showed minimum energy loss during the discharging process. Table 2 lists the specific capacitance, specific energy, and specific power of the MSNT/MnO_2_ asymmetric cell. The long-term cycling stability of the cell was evaluated by obtaining its CV curves at a fixed scan rate of 100 mV s^−1^ for 2000 cycles (Fig. 10e). The MSNT//MnO_2_ asymmetric cell could retain 80% capacitance after 2000 cycles. The effect of cycling on the CV curves of the cell is shown in Fig. 10f. The area of the CV curve decreased with an increase in the number of cycles because of the degradation of the electrode material during cycling. Table 3 summarize the electrochemical performance of the MnO_2_//MSNT asymmetric supercapacitor cell and cells from literature.^7–9,66–76^

**Table 2:** Electrochemical parameters of the MSNT//MnO_2_ asymmetric cell

Current density (A g^−1^)	Specific capacitance (F g^−1^)	Energy density (W h kg^−1^)	Power density (W kg^−1^)
1.5	112	35.6	964
3.8	62	17.8	2347
7.6	34	9.9	4752
11.5	24	6.8	6994
15.3	19	5.7	9327

**Table 3:** Electrochemical performance of the MnO_2_//MSNT asymmetric supercapacitor cell and cells from literature

Cell configuration	Electrolyte	Cell potential (V)	Specific capacitance (F g^−1^)	Energy density (W h kg^−1^)	Stability retention@cycles	Ref.
MnO_2_//MSNT	1 M LiCl	1.6	112	35.6	80%@2000	This work
Ni_2_P//AC	6 M KOH	1.4	96	26	91%@5000	7
NiCo_2_O_4_-rGO//AC	1 M NaOH	1.7	—	12	—	66
NiCo_2_O_4_/AC	1 M NaOH	1.7	—	17.72	100%@2000	67
RuCo_2_O_4_//AC	2 M KOH	1.4	133	32.6	82%@2500	68
PPy-NTs//N-CNT	1 M H_2_SO_4_	1.4	109	28.95	90%@2000	69
Co(OH)_2_//GO	1 M KOH	1.2	59	11.9	—	70
Ni(OH)_2_-CNT//rGO	1 M KOH	1.8	78	35.24	—	71
ZnCo_2_O_4_//AC	3 M KOH	1.6	82	27.78	178%@2000	72
NoCo_2_S_4_//AC	3 M KOH	1.6	72	25.5	86%@4000	73
α-NiMoO_4_//rGO	3 M KOH	1.1	73	12.31	85%@2000	74
NaMnO_2_//AC	0.5 M Na_2_SO_4_	1.9	39	19.5	97%@10 000	75
Co_0.1_Ni_0.9_P//AC	2 M KOH	1.4	119	32.2	72%@10 000	8
FeCo_2_O_4_//AC	6 M KOH	1.4	88	24	94%@5000	76
NiP-CNT//CoP-CNT	2 M KOH	1.4	151	41.1	70%@10 000	9

## 4. Conclusion

In this study, we developed a novel simple, scalable, and efficient approach to prepare composite electrodes with excellent charge storage performance. We synthesized MSNT composite thin films using the SILAR method for supercapacitor applications. MoSe_2_ (lichen-like growth) was deposited on a MWCNT nanonetwork, and the resulting MSNT film showed excellent electrochemical properties such as a specific capacity of 192 mA h g^−1^ and a cyclic stability of 88%. This excellent electrochemical performance of the composite film can be attributed to the large surface area of the MWCNT nanonetwork with a good conductive path and the effective synergistic effect and interfacial conjugation between MoSe_2_ and the MWCNTs. This study provides a new pathway for the synthesis of hybrid carbon- and metal selenide-based thin films for energy storage applications. Inspired by the enhanced electrochemical performance of the MSNT composite electrode, an aqueous asymmetric cell was fabricated using MnO_2_ as the pseudocapacitive positive electrode. The MSNT//MnO_2_ asymmetric cell delivered a high specific capacitance of 112 F g^−1^ with an energy density of 35.6 W h kg^−1^ and a capacitance retention of 80% after 2000 cycles.

## Conflicts of interest

There are no conflicts of interest to declare.

## Supplementary Material

RA-010-D0RA06952C-s001
