# Supplementary material for: Lichen-like anchoring of MoSe2 on functionalized multiwalled carbon nanotubes: an efficient electrode for asymmetric supercapacitors
Source: RSC Adv. 2020 Nov 4;10(66):40092–105. doi: 10.1039/d0ra06952c (PMC9057468; doi:10.1039/d0ra06952c)
Supplement: RA-010-D0RA06952C-s001 [file RA-010-D0RA06952C-s001.pdf]

Electronic Supplementary Material (ESI) for RSC Advances.
This journal is © The Royal Society of Chemistry 2020

Lichen-Like Anchoring of $MoSe_2$ on Functionalized Multiwalled Carbon Nanotubes: An Efficient Electrode for Asymmetric Supercapacitor

Swapnil S. Karade[a], Ajaysing S. Nimbalkar[b,c], Jeong-Hyun Eum[a] and Hansung Kim[a,*]

[a]Electrochemical Energy Laboratory, Department of Chemical and Biomolecular Engineering, Yonsei University, 50 Yonsei-ro, Seodaemun-gu, Seoul-03722, Republic of Korea.

[b]Korea Research Institute of Chemical Technology, Yusong-gu, Republic of Korea.

[c]University of Science and Technology, Daejeon, Republic of Korea.

Supporting information (S1)

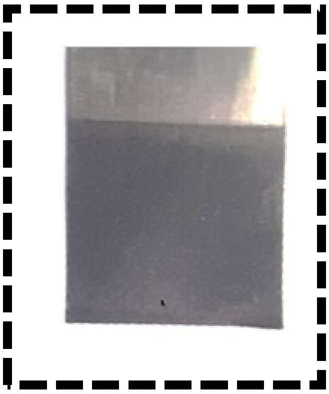

Figure S1: Photograph of MSNT composite deposited thin film

Supporting information (S2)

Characterizations

Structural study of MWCNTs, $MoSe_2$ and MWCNTs/$MoSe_2$ (MSNT) thin films were carried out with an X-ray diffractometer (XRD) using Cu-Kα radiations (wavelength λ = 1.5406 °A) (System Ultima IV of Rigaku Corporation, Japan). The X-ray photoelectron spectroscopy (XPS) analysis was carried out for finding chemical composition and oxidation states of the sample materials using K-alpha (Thermo VG, U.K.) X-ray photoelectron spectrometer microprobe. The film surface morphology was observed by field emission scanning electron microscope (FESEM) from JEOL JSM-6360, Mira-3, Tescan, Brno-Czech Republic.

Supercapacitor electrode study was performed by using the Potentiostat/Galvanostat PARSTAT 4000 (Princeton Applied Research, USA). The mass loading plays an important role in order to evaluate specific electrochemical parameters. In this regard, the weight of MSNT composite thin film electrode was 0.40 mg cm^{-2}, whereas the weight of $MoSe_2$ thin film deposited on stainless steel substrate was 0.23 mg cm^{-2}, respectively.

Calculations

The specific capacity calculated using the following equations

$$C_s\,(C\,g^{-1}) = \frac{\int_{V_i}^{V_f} I\,(V)\,dv}{m\,v} \qquad (1)$$

$$C_s\,(mAh\,g^{-1}) = \frac{\int_{V_i}^{V_f} I\,(V)\,dv}{3.6\,m\,v} \qquad (2)$$

Where, $\int_{V_i}^{V_f} I(V)dV$ is half integral area of the CV curve, m is the mass loading of the active electrode ($MoSe2$ = 0.23 mg cm^{-2} and MSNT=0.42 mg cm^{-2}), and υ is the scan rate.

Specific capacity of the electrode can be calculated using charge-discharge plot with the help of following equation.

$$Q_s(mAh\, g^{-1}) = \frac{I\int Vdt}{3.6\, m\, V} \qquad (3)$$

Specific capacitance (C) from galvanostatic charge-discharge can also be calculated as

$$C\,(F\, g^{-1}) = \frac{I\int Vdt}{mV^2} \qquad (4)$$

where, 'I' implies current intensity, 'm' is the active electrode mass, 'V' is the potential window, and '$\int Vdt$' entails are enclosed only under the discharge curve. For the symmetric and asymmetric cell, the total mass loading of the both the electrodes were used to calculate the specific capacitance.

Further specific energy (E) in W h kg^{-1} associated with specific power (P) in W kg^{-1} of electrode were evaluated from the charge-discharge dimensions using the following equivalence,

$$E = \frac{1}{2}\left[\frac{CV^2}{3.6}\right] \qquad (5)$$

$$P = \frac{3600 \times E}{\Delta t} \qquad (6)$$

where, Δt is discharge time.

Supporting information S3

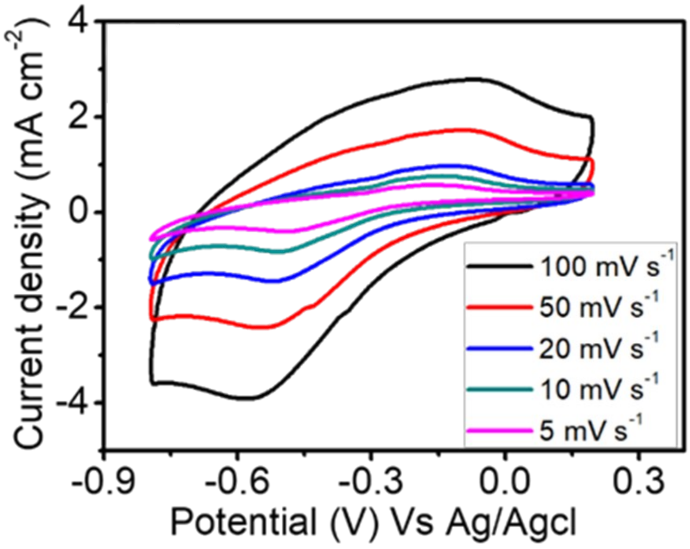

Figure S2: CV curves for $MoSe_2$ electrodes at 5 to 100 mV s^{-1} scan rates.

Supporting information S4

According to the charge balance theory, mass ratio is estimated based on following equation.

$$\frac{m^+}{m^-} = \frac{Q^-}{Q^+} = \frac{C_{s-} \times V_-}{C_{s+} \times V_+} \qquad (7)$$

Where, 'm^+' and 'm^-' are the active electrode mass of positive and negative electrodes respectively, 'Q^+' and 'Q^-' are the charge stored by the positive and negative electrodes, 'C_s^+' and 'C_s^-' are the specific capacitance values of the positive and negative electrodes, and 'V_+' and 'V_-' are the potential window of positive and negative electrodes. The specific capacity (specific capacitance) of 144 mAh g^{-1} (517 F g^{-1}) and 66 mAh g^{-1} (236 F g^{-1}) was calculated for MSNT and MnO_2 electrodes from CV curve. The mass ratio of the positive and negative electrode was balanced at 2.19:1 on the basis of charge balance theory in which specific capacitance values of 236 F g^{-1} and 517 F g^{-1} and voltage window of 0.8 V and 1 V was used for positive and negative electrode respectively.

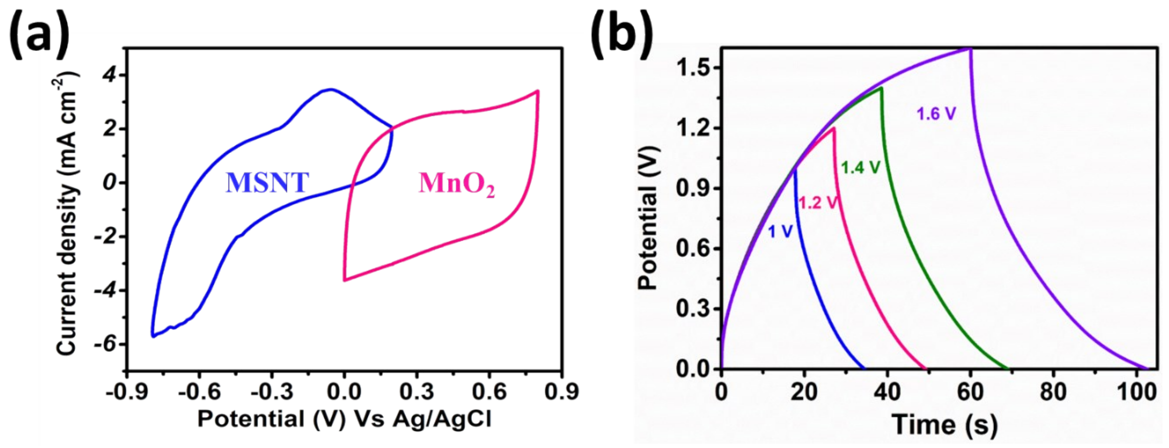

Figure S3: (a) CV curves of MSNT negative and MnO_2 positive electrode measured at 20 mV s^{-1} scan rate and (b) GCD plot of MSNT//MnO_2 asymmetric cell with different potential limit at current density of 2.5 A g^{-1}.
